# Dramatic Resolution of an Unresectable Giant Basal Cell Carcinoma Treated with Intensity-Modulated Radiation Therapy (IMRT) - A Case Report

**DOI:** 10.7759/cureus.416

**Published:** 2015-12-17

**Authors:** Narine Wandrey, Tiffany Chen, Tony Eng

**Affiliations:** 1 Department of Radiation Oncology, Cancer Therapy and Research Center (CTRC), UT Health Science Center at San Antonio

**Keywords:** giant basal cell carcinoma, radiotherapy, intensity modulated radiation therapy

## Abstract

A 59-year-old man presented with an unresectable bulky giant basal cell carcinoma on his upper back. A trial of chemotherapy did not help relieve his symptoms or reduce the tumor. He was referred for and received definitive radiation therapy via IMRT with dramatic regression. The patient had been unable to lie on his back for many years but currently can sleep comfortably on his back without pain, which has dramatically improved his quality of life.

## Introduction

Basal cell carcinoma (BCC) is the most common cutaneous malignancy. Most BCC cases are small, superficial, less than 5 cm in size, and have an indolent course [1]. Because of its high prevalence, numerous well-established modes of treatment are available, including Moh’s micrographic surgery, surgical excision, radiation therapy, photodynamic therapy, or immunotherapy. Variants of BCC exist, most notably giant BCC, which has greater metastatic potential due to its large size and limited treatment options. Giant BCC represents only 0.4 - 1% of all BCC and is defined as a lesion > 5 cm in diameter, most often occurring on the trunk [2-3]. Because giant BCC is a rare malignancy, there are no standard guidelines for treatment, but wide local excision with or without postoperative adjuvant therapy (radiotherapy or chemotherapy) is commonly used [1]. Here, we present a case report on an individual with giant BCC treated with radiotherapy with definitive intent. This case report serves to offer more statistical power on a rare malignancy and definitive radiation treatment as one of the alternative options for the disease.

## Case presentation

A 59-year-old man first noticed a lesion that appeared as a small mole in 1998 on his upper back. The lesion was neglected and continued to grow until it became progressively symptomatic with pain and bleeding in June 2012 when a biopsy confirmed basal cell carcinoma. No regional or distant metastatic disease was noted on his workup (Stage cT2, N0, M0). The lesion was deemed unresectable because of its bulky size (~10 cm) and proximity to the spine, and the patient was deemed a poor surgical candidate due to his past medical history of COPD, CAD, epilepsy, and hypertension. In 2014, he was given an oral chemotherapeutic, Erivedge (vismodegib), for 11 months with no tumor regression. The medication was stopped due to neuropathic side effects and development of seizures. Past surgical history is non-contributory. Family history included two brothers and a sister with basal cell carcinoma, father with stomach cancer, and mother with colon cancer. The patient was a painter for 42 years with reported significant sun exposure, history of alcoholism but no alcohol for two years, and a 70-pack-year history, currently smoking 6 cigarettes per day. He had no history of prior radiation therapy. He had generalized weakness, fatigue, and significant pain in the area of the lesion; he has not slept on his back for over 10 years due to the pain. He denied any fevers, weight loss, or other skeletal pain. Physical exams during his prior visits were pertinent for hypertension, negative lymphadenopathy, and a 10 x 10 cm unroofed bloody lesion approximately 4-5 cm outwardly growing from the back. His CBC, urinalysis, liver function tests, BUN, and creatinine were within normal limits. Imaging studies were not done due to the tumor preventing the patient from lying on his back.

Informed patient consent was obtained. No identifying patient data was disclosed in this report.

### Treatment

**Figure 1:**
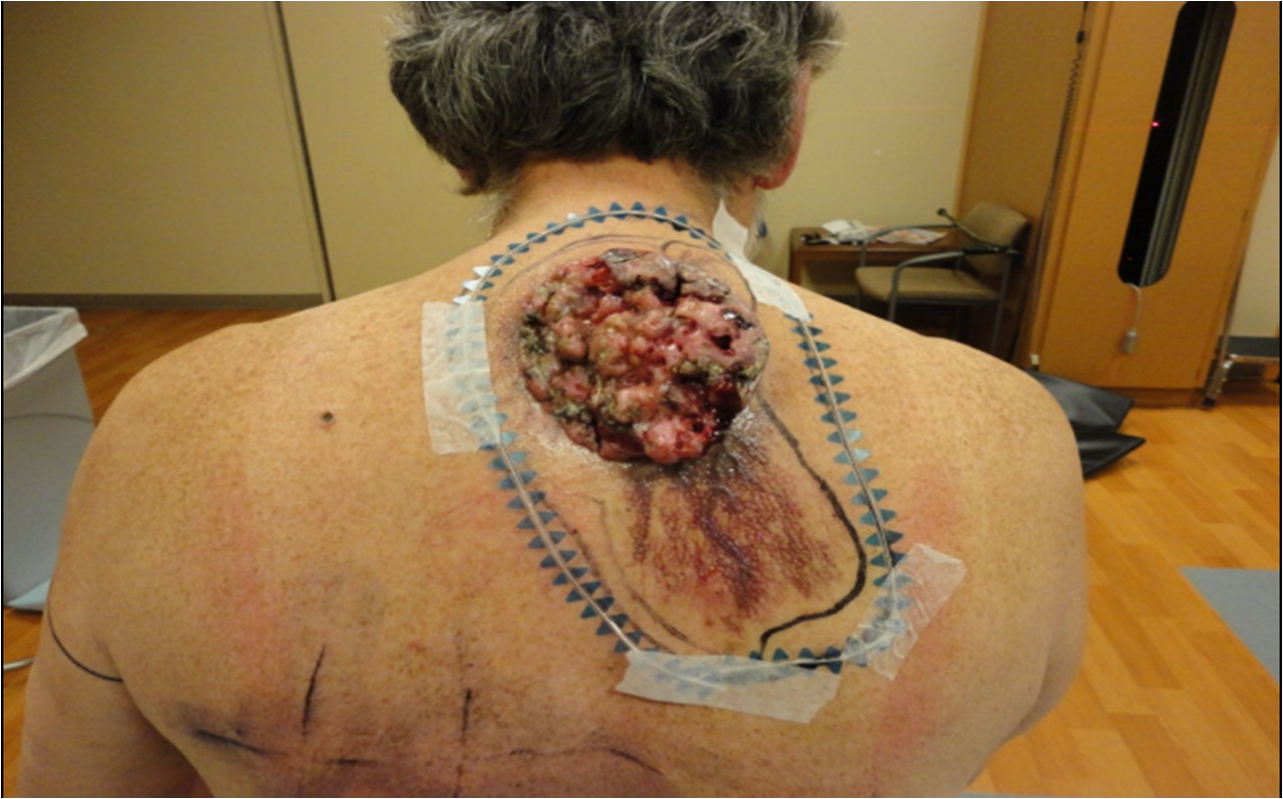
A bulky, friable giant basal cell carcinoma with suspicious subdermal spread inferiorly. The outer wire marks the radiation treatment border.

The patient was referred to radiation oncology for treatment evaluation in February 2015. At the time, the tumor was noted to be a friable, fixed, deeply seated, fungating bulky mass in the upper back over the spine, 10 x 12 cm (base) x 5 cm (height) in size with an adjacent area of suspicious discolored subdermal spread inferiorly (Figure 1).

**Figure 2:**
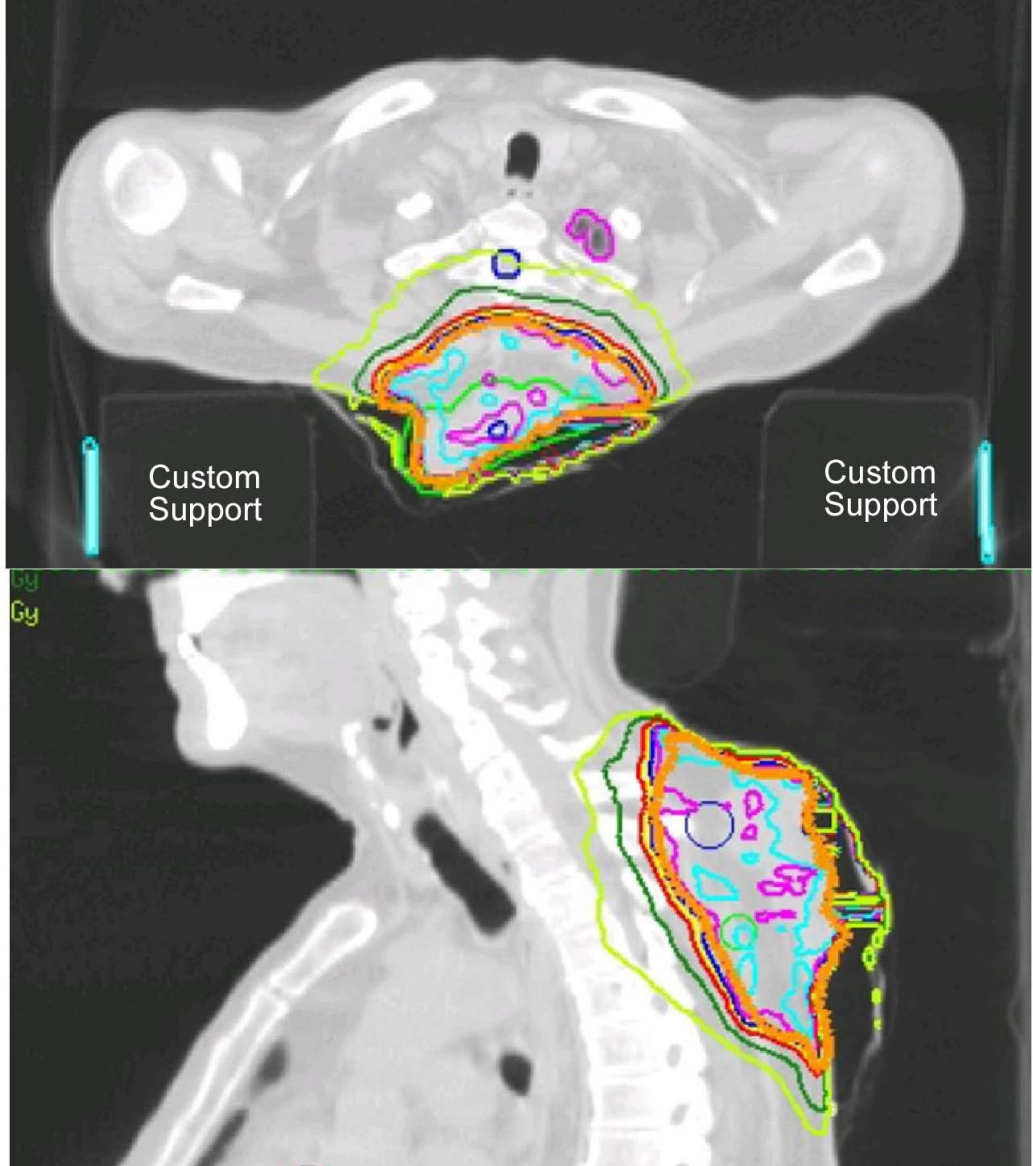
IMRT treatment plan orange-planning target volume; blue-100% isodose line; red-90% isodose line; dark green-70% isodose line; light green-50% isodose line.

Clinically, no regional adenopathy or distant metastasis was noted on exam or simulation CT with custom back support for radiation treatment planning. However, the lesion was noted to be approaching the adjacent spinous processes of upper thoracic vertebrae (Figure 2).

**Figure 3:**
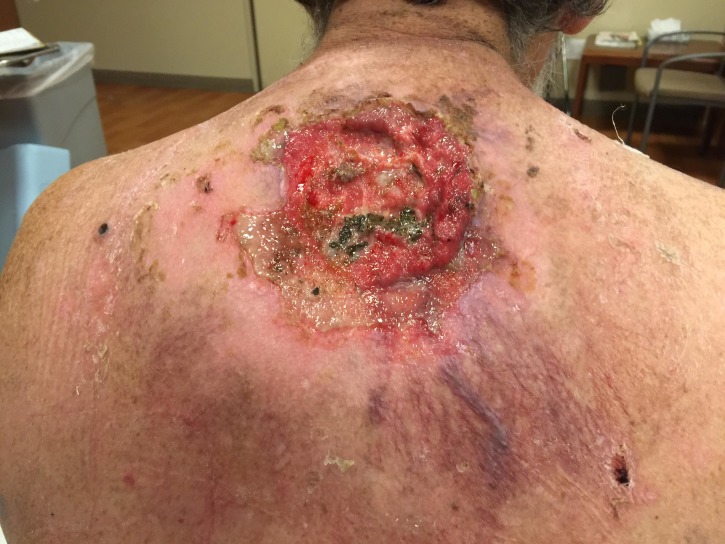
Dramatic resolution of the bulky giant basal cell carcinoma at completion of external beam radiotherapy.

He received a total dose of 60 Gy in 30 daily fractions via IMRT with 6 MV external photon beam and, as the tumor shrunk, a 20 Gy boost in 10 daily fractions with 12 MeV and 9 MeV superficial electron beam therapy, with a 1 cm bolus to improve surface dose. He tolerated radiotherapy well without significant side effects or complications. Treatment was started in February 2015 and completed in May 2015 with dramatic regression and pain relief (Figure 3).

**Figure 4:**
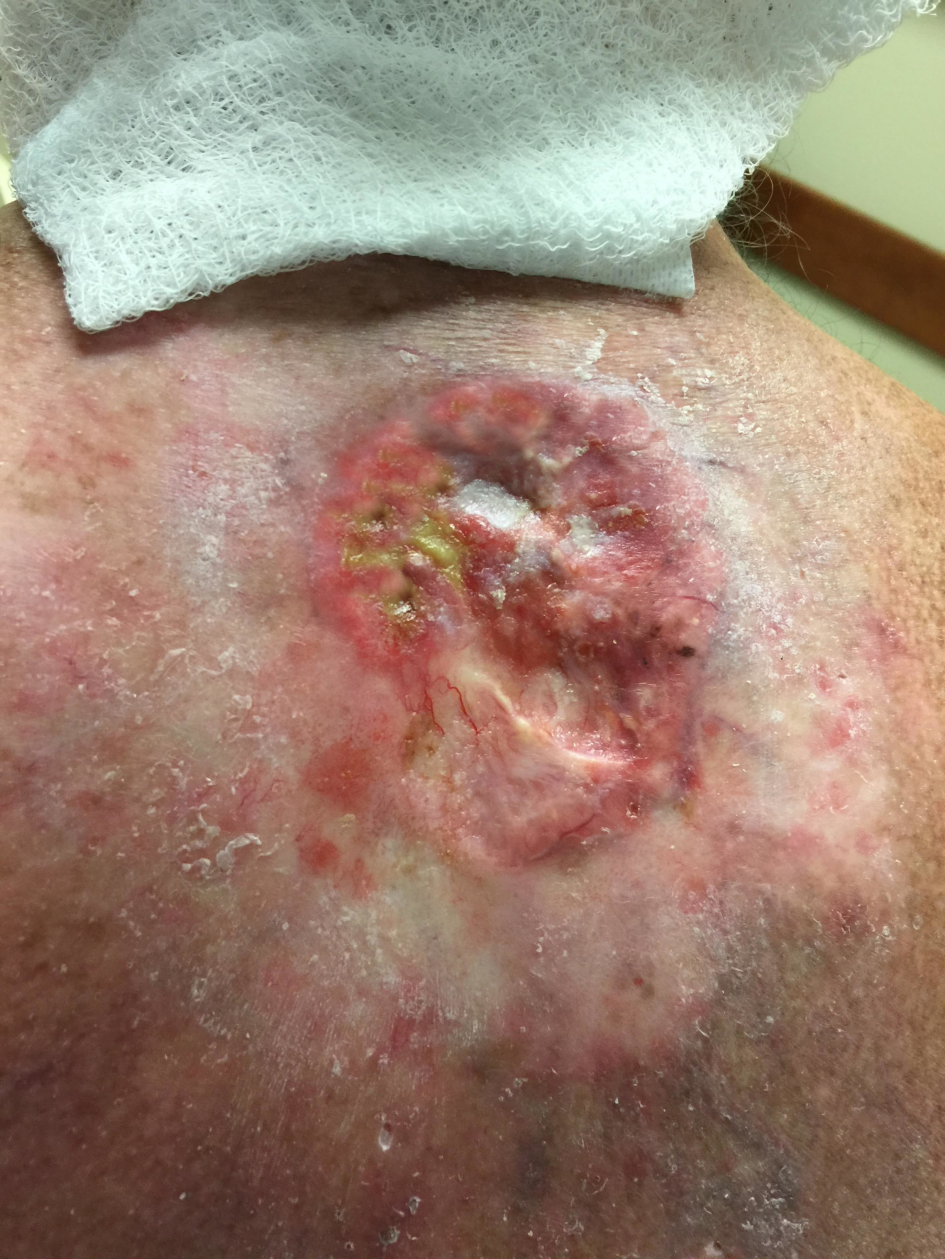
Healing wound with granulating tissue from the edge towards the center five months after radiotherapy. Suspicious subdermal spread has mostly faded away.

The patient stated that he was able to sleep on his back for the first time in over 10 years. On his last follow-up in October 2015, continued resolution of the mass with granulating tissue was noted (Figure 4). The lesion appeared as a healing wound with a small residual area of shrinking fleshy-appearing center, approximately 2-3 cm in size with irregular borders. A follow-up diagnostic CT scan of the chest to assess disease status showed only minimal irregularity within subcutaneous soft tissues of the upper back and several subcentimeter right upper and lower lobe pulmonary nodules interpreted as likely prior granulomatous disease. The patient was scheduled to return in three months for next follow-up.

## Discussion

Unlike common BCC, the etiology of giant BCC is not entirely understood. While small superficial BCC is mostly caused by UV radiation and may be associated with diseases, such as xeroderma pigmentosum and albinism, giant BCC is thought to be due to neglect of initial disease [4], as seen in this patient, who had no specific social issues but apparently did not like to see physicians until he was in significant pain. Since giant BCC is a rare oncological entity, treatment options are not well elucidated. Historically and intuitively, based on anecdotal cases, giant BCCs have been treated with wide local excision, while the risk of tumor residue is relatively high [1]. Multi-modality therapy has also been advocated because of a higher potential of metastatic disease at initial presentation. In a literature review, metastasis was found in 17.6% of patients on initial presentation [1]. The authors suggested the optimal management of giant BCC to be wide local excision with histologically confirmed tumor-free margins, followed by adjuvant therapy, and lymphadenectomy when necessary. However, many patients are not surgical candidates, including the patient in this case report, and, therefore, require different treatment approaches. Treatment with topical 5% imiquimod two to three days/week for 12 weeks resulted in two giant BCC cases being disease-free at six years and 3.5 years follow-up [5]. Erivedge (vismodegib), a chemotherapeutic, acts as a cyclopamine-competitive antagonist of the smoothened receptor and prevents the expression of tumor-mediating genes within the hedgehog pathway in more than 90% of basal-cell carcinomas. It has also been reported as a successful treatment in giant BCC for some non-surgical candidates [6]; however, it did not make a significant clinical impact on this patient. A combination of 5% imiquimod and cryosurgery has also been used on a patient with giant BCC on the scalp [7].

Radiobiologically, indolent bulky tumors rarely show a dramatic clinical response to external beam therapy because of hypoxia and low mitotic activity. Therefore, external beam radiotherapy, which is very effective in treating small skin cancers, is often not the first line therapy for giant BCC [8]. To our knowledge, this may be the first case of an unresectable bulky giant BCC in the upper back successfully treated with high-dose definitive external beam radiotherapy using IMRT. We acknowledge that the follow-up time is still short. Although he presented with no regional or distant metastasis at the time of treatment evaluation, the prognosis of his disease remains uncertain. As the incidence of BCC is increasing every year at a rate of 3%, and giant BCC is a variant of BCC, we would expect the incidence of this malignancy to rise [9]. Understanding treatment options for patients with giant BCC will aid the increasing numbers of diagnosed patients. Further studies are definitely needed to define other independent prognostic variables, such as patient age, histology of tumor, and site of disease and to determine the optimal therapy in patients with giant BCC.

## Conclusions

There is no definitive consensus on treatment for giant BCC, although surgical wide excision with adjuvant therapy and lymphadenectomy when necessary has been successful in some patients. When surgery is not possible, definitive radiation therapy and/or chemotherapy are alternative therapeutic options. This case report highlights the dramatic regression of an unresectable giant BCC using IMRT only. The patient had been unable to lie on his back for many years but currently can sleep comfortably on his back, which has dramatically improved his quality of life. Due to potential greater rates of metastasis in giant BCC compared to that of small superficial BCC, this patient will need close follow-up. Further studies should be conducted to determine the optimal treatment for patients with giant BCC.
